# Unusual Profile of Germline Genetic Variants in Unselected Colorectal Cancer Patients from a High-Prevalence Region in Panama

**DOI:** 10.3390/genes16080890

**Published:** 2025-07-28

**Authors:** Iván Landires, José Pinto, Raúl Cumbrera, Alexandra Nieto, Gumercindo Pimentel-Peralta, Yennifer Alfaro, Virginia Núñez-Samudio

**Affiliations:** 1 Unidad de Genética y Salud Pública, Instituto de Ciencias Médicas, Las Tablas 0710, Panama; raulcumbrera@gmail.com (R.C.); gumercindopimentel@gmail.com (G.P.-P.); yennyissel14@gmail.com (Y.A.); 2 Consulta de Genética Médica, Hospital Joaquín Pablo Franco Sayas, Región de Salud de Los Santos, Ministry of Health, Las Tablas 0710, Panama; 3 ICM LAB, Las Tablas 0710, Panama; 4 Unidad Oncológica de Azuero, Hospital Regional Anita Moreno, Región de Salud de Los Santos, Ministry of Health, Las Tablas 0710, Panama; josepintollerena@gmail.com (J.P.); alexandra.nieto28@hotmail.com (A.N.); 5 Sección de Epidemiología, Departamento de Salud Pública, Región de Salud de Herrera, Ministry of Health, Chitre 0601, Panama

**Keywords:** germline variants, colorectal cancer, Panama

## Abstract

Background: The profile of germline genetic variants among colorectal cancer patients in Panama has not yet been explored. Methods: We recruited 95 patients with colorectal cancer in an Oncology Reference Hospital Unit in the Azuero region of central Panama, which exhibited the highest prevalence of colorectal cancer in Panama. DNA analysis was performed with a panel of 113 genes with germline mutations for cancer (TruSight^®^ Cancer Sequencing Panel from Illumina, San Diego, CA, USA). Results: Among the 95 cases, 10 pathogenic/likely pathogenic variants (P/LP) were identified in the MUTYH, TP53, CHEK2, PALB2, ATM, and BARD1 genes, representing 10% of the total. The variant 1103G>A (p.Gly368Asp) in MUTYH was the most prevalent. The variant at c.1675_1676delCAinsTG (p.Gln559Ter) in PALB2 is new and is reported for the first time in this study. Variants were most frequently detected in the MUTYH and CHEK2 genes, affecting four and two patients, respectively. Notably, none of the 95 Panamanian patients in the initial colorectal cancer cohort had mutations in mismatch repair (MMR) genes. These genes are among the most frequently mutated in other cohorts around the world. Conclusions: The atypical profile of germline genetic variants in this population may be related to the unique characteristics of the Azuero population in Panama’s central region. This profile may partly explain the high prevalence of colorectal cancer among its inhabitants.

## 1. Introduction

According to the World Health Organization (WHO), colorectal cancer is the third most prevalent cancer and the second leading cause of cancer-related deaths worldwide [1]. Globally, colorectal cancer is the second most frequent cause of Disability-Adjusted Life Years (DALYs) being lost due to cancer for both sexes combined [2].

All types of cancer are the second leading cause of death in Panama. Nationwide, colorectal cancer is the fourth leading cause of morbidity and mortality among all cancers. In the Azuero region, which includes the provinces of Herrera and Los Santos, as well as the southernmost part of the province of Veraguas, colorectal cancer is the second most prevalent cancer and the leading cause of cancer-related deaths [3]. Because of its higher prevalence and mortality rate in the Azuero region compared to the rest of the country, it is important to conduct studies to determine why colorectal cancer is so prevalent in the Azuero region of Panama.

According to the 2023 census, Panama has a population of 4.4 million. Of those, 31.7% are of African descent, 17.2% are indigenous, and 51.2% are mestizo [4]. The population of the Azuero region has unique ethnic and cultural characteristics within the Republic of Panama. Due to the Spanish conquest and colonization, the region is currently considered one of the most Hispanicized in Panama. The region is also distinguished by a culture that blends Spanish, Amerindian, and African traditions, resulting in the distinctive Azuero culture of today [5]. Recent studies on the proportion of mitochondrial DNA [6] and Y chromosomes [7] in different regions and provinces of Panama indicate that, although the percentage of mitochondrial DNA of Native American origin exceeds 80% in all Panamanian regions, with less African and Eurasian influence, the Azuero region has the highest proportion of Y chromosomes of Eurasian origin in the country, exceeding 75%. This indicates that maternal ancestry, determined by mitochondrial DNA, is Amerindian in Panama and in the Azuero region, while paternal ancestry in the latter was mainly Spanish. This highlights that the process of Spanish conquest and colonization led to a more Hispanicized mestizaje in Azuero than in the rest of Panama’s regions. For this reason, we propose that certain genetic determinants of colorectal cancer predisposition may be characteristic of Azuero’s inhabitants.

Twenty-five to thirty percent of colorectal cancer cases are related to non-modifiable risk factors, such as genetic predisposition, personal history of polyps or adenomas, and family history of colorectal cancer. Examples of these factors include Lynch syndrome and familial adenomatous polyposis [2]. Genetic studies have identified heterozygous pathogenic and/or likely pathogenic variants (PVs/LPVs) in mismatch repair (MMR) genes as the most frequent genetic cause of colorectal cancer. These variants are associated with Lynch syndrome, also known as hereditary nonpolyposis colorectal cancer (HNPCC). MMR is essential for repairing DNA mismatches during replication. When the MMR mechanism is defective, somatic mutational events accumulate in genes with tandem repeats of DNA called microsatellites. Monoallelic APC gene PVs and the biallelic inactivation of MUTYH are frequent causes of hereditary adenomatous polyposis and colorectal cancer [8,9,10].

This study presents an unusual profile of germline genetic variants that predispose individuals to colorectal cancer. The predominant genes in this profile are MUTYH, CHEK2, TP53, PALB2, ATM, and BARD1. This genetic profile may be related to the distinctive characteristics of the mestizo population in Azuero, a region in central Panama. It may also partially explain the region’s high prevalence of colorectal cancer. To our knowledge, this is the first study to evaluate germline variants of genetic predisposition to colorectal cancer in Panamanian patients.

## 2. Materials and Methods

We conducted a study of patients with colorectal cancer unselected for family history or age of onset and undergoing treatment and follow-up at the Unidad Oncológica de Azuero from Hospital Regional Anita Moreno de Los Santos between March 2024 and March 2025. This hospital is the primary referral center for cancer diagnosis and treatment in Panama’s central region.

To be enrolled in this study of germline genetic variants in patients diagnosed with colorectal cancer recruited without selection criteria, patients had to be at least 18 years of age, mentally competent, and willing to sign informed consent documents, regardless of sex. All 95 patients were diagnosed at the Unidad Oncológica de Azuero from Hospital Regional Anita Moreno de Los Santos. All patients voluntarily agreed to participate and provided written informed consent. Blood samples were collected with the approval of the Bioethics Committee of Chicho Fábrega Hospital (study number CBI-HRLCHF-EC-CBIHRLCHF-2023-08102).

Family histories of cancer were collected for each patient using three-generation family trees.

Ten milliliters (10 mL) of blood were drawn from the forearm vein of consenting participants using an EDTA tetrasodium anticoagulant. The samples were briefly preserved at 4 °C before genomic DNA was extracted using a QIAamp^®^ DNA Mini Kit (Qiagen, Hilden, Germany). The DNA concentration was measured using a Qubit 4™ Fluorometer (Thermo Fisher Scientific, Waltham, MA, USA). DNA samples with a concentration above 1 μg were used to prepare sequencing libraries with the TruSight Hereditary Cancer Panel to identify germline mutations across 113 genes. The libraries were sequenced on a MiniSeq (Illumina, Inc., San Diego, CA, USA) according to the manufacturer’s protocols. We mapped all high-quality data to the human genome assembly using the bwa-mem 2 algorithm. Next, we used the Genome Analysis Toolkit (GATK 4) to recalibrate base quality, realign indels, and remove duplicates. According to GATK best practices, we performed single-nucleotide polymorphism (SNP) and indel discovery and genotyping. Next, we applied quality score recalibration and filtering to all variant calls to remove low-quality variants. We analyzed the genetic data using Golden Helix^®^ SVS 8.8.3 (Bozeman, MT, USA) and performed quality control as recommended in previous studies. We filtered variants for missense or frameshift mutations, stop gains or losses, initiator codons, in-frame insertions or deletions, and splice site alterations. Variants with a minor allele frequency < 0.25 were excluded from analysis. We evaluated the predicted effect of the variants using ClinVar database [11].

## 3. Results

A total of 95 unselected colorectal cancer patients were tested for variants in cancer predisposition genes using the TruSight Hereditary Cancer Panel. On average, 2.3 years passed between diagnosis and the date of the interview and blood sample collection. The median age at diagnosis was 67 years, and the median age at the time of the interview was 70 years. A total of 7 percent of patients were diagnosed before age 50, and 28.4% were diagnosed before age 60.

**Figure 1:**
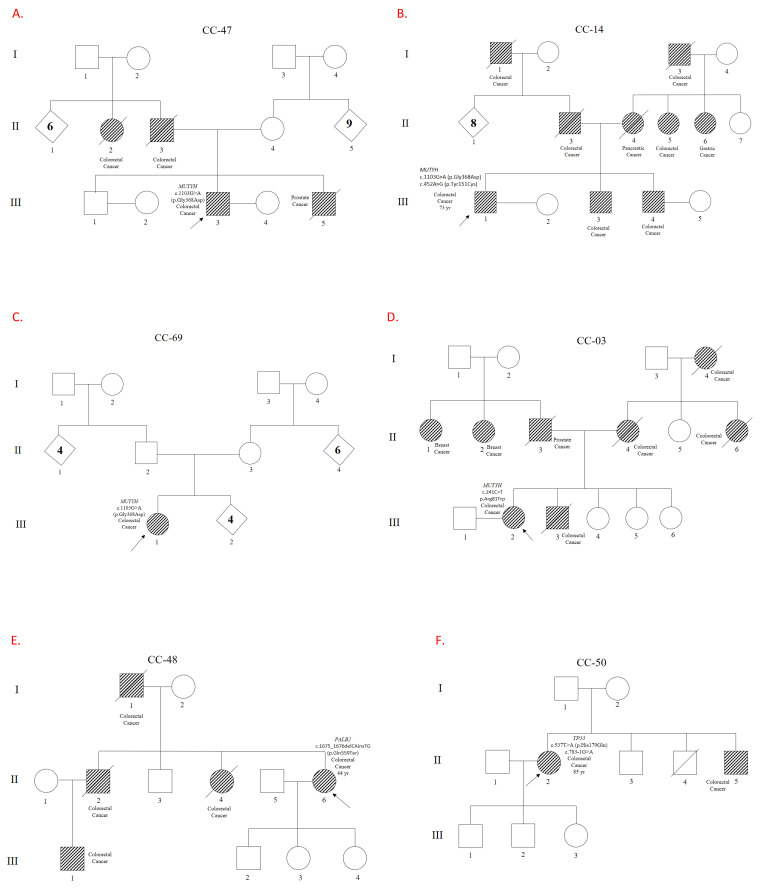

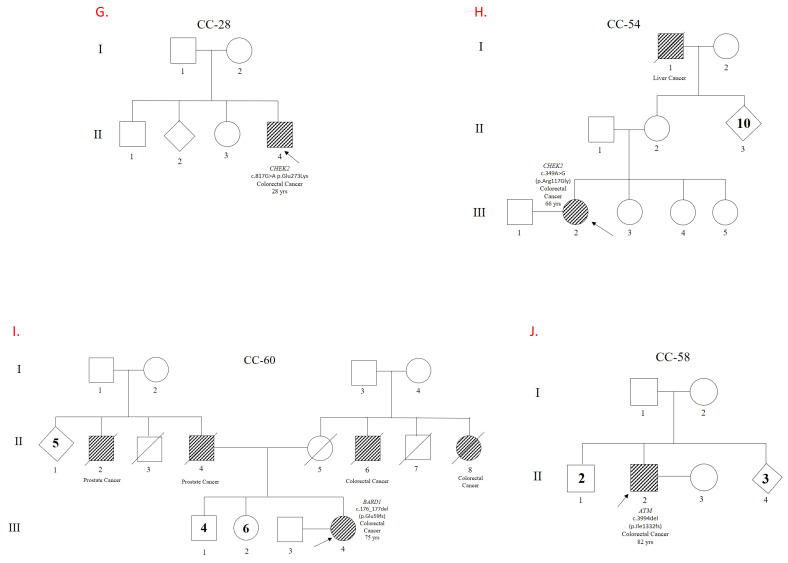
Genealogical trees showing families and patients carrying Pathogeic/Likely Pathogenic variants. Pedigrees of the patients and their families are shown. Panels (**A**–**D**) illustrate variants in the MUTYH gene. Panel (**E**) shows a variant in PALB2, and Panel (**F**) in TP53. Panels (**G**,**H**) depict variants in CHEK2, while Panels (**I**,**J**) correspond to variants in BARD1 and ATM, respectively. Codes beginning with ‘CC’ refer to individual patients and their families. The relevant gene and variant descriptions can be found next to each patient symbol.

A P/LP variant was found in 10 out of 95 patients (10.5%) (Figure 1). Of these patients, four had a variant in MUTYH (4.2%), two had a variant in CHEK2 (2%), one had two variants in TP53 (1%), one had a variant in ATM (1%), one had a variant in BARD1 (1%), and one had a variant in PALB2 (1%) (see Table 1). One patient had two P/LP variants in MUTYH: p.Gly368Asp and p.Tyr151Cys. Another patient had the p.Arg81Trp variant in MUTYH. The p.Gly368Asp variant was the most prevalent MUTYH variant, occurring in three out of four patients with MUTYH variants. The second gene with the highest frequency of P/LP variants was CHEK2: two patients had the p.Glu273Lys and p.Arg117Gly variants with respective ages at onset ranging from 41 to 45 and 61 to 65 years. Neither patient with deleterious CHEK2 variants had a history of affected first-degree relatives, but both had affected second-degree relatives. A male patient with two TP53 variants was also identified: c.783-1G>A, which affects splicing, and a missense variant, p.His179Gln. A patient with a p.Ile1332fs frameshift variant in ATM and another patient with the p.Glu59fs variant in BARD1 were identified. This study reported the p.Gln559Ter variant in PALB2 for the first time in a young patient with an age of onset between 31 and 35 years and with a family history of cancer in first- and second-degree relatives.

Twenty percent of patients with P/LP variants were diagnosed before turning 50, compared to 3.5% of patients without them (*p* = 0.08). Of those diagnosed under 50, 40% had P/LP variants, compared to 9% of those diagnosed over 50 (*p*= 0.08).

Five out of ten patients with a P/LP variant had a first-degree relative with cancer (50%; see Figure 1 and Table 1) 33 out of 85 patients without a P/LP variant had a first-degree relative with cancer (38%) (*p*= not significant). The other cancers present in the ten families with a P/LP variant included three cases of pancreatic cancer, and one case each of breast, prostate, and liver cancer.

## 4. Discussion

Germline genetic variants have been studied in 95 patients with colorectal cancer from a highly prevalent region in Panama. Concerning the frequency of variants within the total population of patients examined, 10.5% were identified as P/LP variants with an atypical profile. Of these, 4% corresponded to patients with variants in the MUTYH gene, 2% in CHEK2, 2% in TP53, and 1% for each of the genes in ATM, BARD1, and PALB2. No patients with germline variants in the MMR genes were found; nevertheless, these genes have been identified as the most frequently mutated genes in other colorectal cancer studies.

To our knowledge, this is the first study to evaluate germline genetic variants associated with colorectal cancer in the Panamanian population. This study was conducted in Azuero, a region in central Panama with the highest disease prevalence in the country. Unlike the rest of Panama, Azuero has a significant Spanish mestizo population, in contrast to the rest of the country, where the contribution of Amerindian and African descendants is higher [5]. This could explain, in part, why this population has genetic determinants that cause high rates of colorectal cancer. Our results indicate that approximately 10.5% of patients diagnosed with colorectal cancer in Panama have pathogenic or likely pathogenic variants in cancer predisposition genes. These observations highlight the importance of investigating the genetic basis of this disease in our population.

The most frequent finding was P/LP variants in the MUTYH gene, identified in four patients. The most prevalent variant in the MUTYH gene was p.Gly368Asp, which was found in two patients in a monoallelic presentation. In a third patient, p.Gly368Asp and p.Tyr151Cys were found in a probable biallelic presentation. A fourth patient had the rare p.Arg81Trp variant in a monoallelic presentation. The p.Gly368Asp variant’s identification as the most prevalent variant is significant because it aligns with previous studies that identified this variant as being prevalent in the Eurasian population. The p.Tyr151Cys variant has also been reported to be significant in terms of frequency among patients with MUTYH mutations and colorectal cancer [12]. Three of the four patients with P/LP variants in MUTYH had a family history of cancer. Up to 2% of people of Northern European descent carry a pathogenic variant in MUTYH [12]. In our unselected patient series, up to 4% were carriers of P/LP variants in MUTYH, highlighting the importance of this marker in germline genetic evaluations of patients with colorectal cancer in Panama. One patient presented with two MUTYH variants in a probable biallelic presentation, while the other three patients presented with one MUTYH variant in a monoallelic presentation, suggesting that these monoallelic variants may have high penetrance in our population. Previous studies have demonstrated a significant correlation between MUTYH-associated polyposis (MAP) and colorectal cancer, as well as with biallelic germline pathogenic variants. A twofold increase in risk has been observed in monoallelic carriers with a family history of cancer. MUTYH is a base excision repair (BER) gene that is involved in repairing oxidative damage. The results of the present study are consistent with a recent study examining germline variants in the MUTYH gene in Latin American patients with colorectal cancer [12]. In that study, the most frequent variant was the p.Gly368Asp mutation. Notably, two out of three patients with a monoallelic presentation in this study had a family history of cancer. These results underscore the importance of family studies for genetic counseling and cancer prevention interventions with relatives who test positive for these variants, even in cases of monoallelic presentation.

The second most frequent gene with P/LP variants was CHEK2, which affected two patients (2%). One patient had the p.Glu273Lys variant and experienced early-onset disease between the ages of 41 and 45. The other patient had the p.Arg117Gly variant and experienced disease onset between the ages of 61 and 65. Neither patient had a history of cancer among their first-degree relatives, though they did have affected second-degree relatives. Patients with CHEK2 P/LP variants in our sample population experienced a relatively early onset of colorectal cancer. This suggests that the CHEK2 gene may play a role in cases of early-onset colorectal cancer in our population. CHEK2 is a cell-cycle checkpoint regulator gene, and research has shown that it plays a pivotal role in DNA repair [13]. However, the role of the CHEK2 gene in predisposition to colorectal cancer remains controversial. Some studies have found a slight association between certain CHEK2 mutations and an increased risk of colorectal cancer. However, the role of the CHEK2 gene in colorectal cancer predisposition remains controversial. Some studies suggest that this relationship may not be significant [13]. For instance, the 2024 National Comprehensive Cancer Network (NCCN) guidelines state that CHEK2 variants are not linked to an elevated risk of colorectal cancer. The guidelines recommend considering other factors to assess individual risk. Nevertheless, the fact that both patients in our cohort with P/LP variants in CHEK2 are younger than the mean age of diagnosis in this cohort (67 years) and both have a family history of the disease suggests that CHEK2 may predispose the Panamanian population to colorectal cancer [14].

An elderly male patient with an age of onset between 81 and 85 years and no family history of cancer was found to have two variants in the TP53 gene. One variant, c.783-1G>A, is likely pathogenic, while the other, p.His179Gln, is pathogenic. These variants were predicted to affect splicing and cause a missense mutation, respectively. Germline mutations in the TP53 gene cause Li–Fraumeni syndrome, a familial cancer condition of diverse tumors, indicating the tumor-suppressor role of p53 in a variety of tissues [15]. Several studies have identified TP53 as a predisposition gene for colorectal cancer [16]. The absence of a family history of cancer and the patient’s advanced age suggest that these variants have reduced penetrance, meaning they may not always lead to cancer. Alternatively, modifying factors may have delayed the onset of colorectal cancer in this patient. These findings underscore the difficulty of genotype–phenotype correlation in TP53 mutations and highlight the importance of considering additional factors when assessing oncologic risk.

A male patient with an age of onset between 76 and 80 years and no family history was found to have the pathogenic variant p.Ile1332fs in the ATM gene. This variant is predicted to cause a frameshift mutation. This makes him an interesting case for evaluating genetic predisposition to colorectal cancer. The ataxia telangiectasia (ATM) mutation is well-documented for its role in repairing DNA double-strand breaks within the DNA damage response (DDR) pathway. Pathogenic mutations in this gene can compromise genomic stability and increase the risk of various malignancies [17]. The p.Ile1332fs mutation introduces a change in the reading frame that leads to nonsense-mediated decay (NMD). It can be hypothesized that frameshift variants, such as the one carried by this patient, may be more prevalent than other types of variants in ATM in patients with colorectal cancer. This could explain the development of the colorectal cancer phenotype in this patient, who has no significant family history and is likely a de novo variant carrier.

A p.Glu59fs variant in the BARD1 gene was identified in a female patient diagnosed with colorectal cancer between the ages of 71 and 75. She had a family history of other neoplasms among her first- and second-degree relatives. The BARD1 and BRCA1 proteins form a heterodimer with multiple tumor-suppressing functions related to DNA repair and apoptosis. Pathogenic BARD1 variants have been shown to moderately increase the risk of developing triple-negative breast cancer [18]. Recent studies have also shown that BARD1 variants may play a role in colorectal cancer in families with a history of the disease [19]. The patient reported in this study has a family history of prostate and renal cancer in a first-degree relative and colorectal cancer in a third-degree relative. These findings suggest that the identified variant may be responsible for her colorectal cancer and the neoplasms in her family.

An unexpected and novel heterozygous variant in PALB2 (rs587780206), p.Gln559Ter, has been identified. This nonsense variant has not been reported in disease-related databases such as the Human Gene Mutation Database (HGMD) or ClinVar, nor in population databases such as gnomAD SVs v2.1. Identification of the variant chr16-23634870-TG-CA (PALB2: p.Gln559Ter) in an individual with a family history of colorectal cancer suggests that this genetic alteration predisposes individuals to the disease. The variant is characterized by a nonsense mutation that introduces a premature stop codon in exon 4 of 13 of the PALB2 gene, resulting in NMD. Specifically, it is a two-base substitution that changes TG to CA at chromosomal positions 1675 and 1676 (see Table 1). This substitution shares a position with a known pathogenic variant involving a single G-to-A change at position 1675. This change has the same effect on translation, resulting in p.Gln559Ter (rs1555461154). According to the ACMG criteria, the novel variant reported in this study is classified as pathogenic based on PVS1, since it is a nonsense loss of function (LOF) variant that is predicted to cause NMD. LOF of the PALB2 gene is known to cause disease, and 1340 LOF variants of this gene have been reported as pathogenic in dbSNP [20]. The variant also meets the ACMG PM2 criterion since it was not identified in the gnomAD database [21]. The absence of the Chr16-23634870-TG-CA variant in genetic databases and the existing scientific literature indicates that it is novel. Together with the patient’s clinical presentation, this underscores the importance of including the variant in future studies evaluating its role in colorectal cancer predisposition. PALB2 protein interacts with BRCA1 and BRCA2 in response to DNA damage and serves as a linker between BRCA1 and BRCA2, which is necessary for BRCA2-mediated homologous recombination repair. Therefore, it is understood that the genes BRCA1, BRCA2, and PALB2 are key cancer susceptibility genes. They function together in the same DNA damage response pathway [22]. Although PALB2 mutations are primarily associated with an increased risk of other cancers, such as breast cancer, recent studies have examined their potential role in hereditary colorectal cancer [23].

Notably, data from this study revealed a higher prevalence of P/LP variants among patients diagnosed under the age of 50 (33% compared to 8% among those aged over 50]. Furthermore, 20% of patients with P/LP variants were diagnosed before the age of 50, compared to just 3.5% of patients without these variants. The analysis of these data indicates that individuals with P/LP variants are more likely to develop colorectal cancer before age 50, compared to those over 50. These findings are consistent with previous studies indicating that individuals diagnosed at a younger age are more likely to carry genetic variants that predispose them to the condition. This underlines the importance of earlier genetic surveillance in younger individuals [23].

This study suggests that patients with P/LP variants and a family history of cancer are more likely to develop colorectal cancer. A total of 50 percent of patients with P/LP variants reported having a first-degree relative with cancer, compared to 38 percent of patients without these variants. These results support the idea that a family history of cancer is an important factor in determining colorectal cancer risk [24].

The results of this study are consistent with those reported in diverse populations worldwide, in which germline genetic variants are found to occur at a frequency of 5–15% among colorectal cancer patients. However, the specific variants may differ depending on the ethnic characteristics of the population studied. This highlights the need for further studies in specific Latin American populations, where genetic characteristics may influence the results [12].

One of the most notable findings of this study was the absence of pathogenic variants in key mismatch repair (MMR) genes such as MLH1, MSH2, MSH6, and PMS2. These genes are commonly found in other international cohorts of colorectal cancer patients, which represents a significant difference compared to other populations. Colorectal cancer associated with pathogenic variants in MMR genes, which predispose individuals to the disease, is one of the most common forms of hereditary colorectal cancer. These variants are responsible for Lynch syndrome, which affects approximately 2–3% of all colorectal cancer cases worldwide, including in Latin America [25]. The lack of variants in the MMR genes within the Panamanian population carries significant implications. First, it suggests that defects in DNA repair may not act as a predisposing factor for colorectal cancer in Panama, unlike other populations. This discrepancy raises an important question about the specific genetic mechanisms that may be involved in the development of colorectal cancer in Panama and indicates that other genetic factors, such as the variants identified in this study, hold greater significance in our population. The absence of variants in the MMR genes could imply that diagnostic protocols and prevention strategies based on genetic testing of these genes are ineffective for this specific population. Therefore, when evaluating germline variants, it would be beneficial to include other relevant genes, such as MUTYH, CHEK2, TP53, ATM, BARD1, and PALB2, given that they are atypical in other populations worldwide yet prevalent in our cohort. This atypical profile of germline variants may be related to the unique characteristics of the Azuero population in central Panama and could explain the high prevalence of colorectal cancer among its inhabitants.

Given the high prevalence of colorectal cancer in Azuero, more extensive research involving larger groups of people is needed to improve our understanding of the impact of genetic variants and environmental factors on this condition. It will be crucial to create specific genetic databases for the Panamanian population as a whole, particularly for those at high risk of colorectal cancer, in order to develop personalized prevention and treatment strategies.

Furthermore, this study highlights the need for more research into the prevalence of colorectal cancer-predisposing variants in Latin American populations, as their distinct genetic profile may affect this prevalence. Identifying new or rare genetic variants in genes such as PALB2 highlights the importance of investigating the role of new variants and predisposition genes in colorectal cancer.

It is important to recognize that this study has limitations due to its relatively small sample size. The study’s limitations also include potential selection or referral bias associated with analyzing data from a single specialized institution, and the likely exclusion of some patients with Lynch syndrome who have unknown MLH1 methylation status, as some sporadic cases of Lynch syndrome may be due to silencing by the hypermethylation of the MLH1 promoter. Although this study focused on the Azuero region, where colorectal cancer rates are the highest in Panama, it should be noted that the genetic predisposition to colorectal cancer in Azuero may differ from that in other regions of Panama and Latin America. The 2.3-year interval between the onset of the disease and the inclusion of patients in this study could have introduced a survival bias, especially if there had been a significant change in the healthcare provided to cancer patients in the oncology unit where the patients were recruited during this time. However, the claim lacks empirical evidence to support it. The latter could favor the overrepresentation or underrepresentation of certain deleterious variants, which would result in the unusual pattern of variants found in this study. Furthermore, despite the extensive gene panel used, other relevant genetic variants may have been overlooked. Further research involving a broader range of genes and a larger number of patients from various regions is therefore required to investigate the relationship between genetic variants and colorectal cancer in Panamanian and Latin American populations.

Beyond their limitations, this work presents novel and relevant evidence regarding the germline genetic variant profile of colorectal cancer patients from the Azuero region of Panama. This initiative marks a pioneering endeavor in the field, offering insights that could inform future genetic screening and personalized medicine practices, particularly among underrepresented populations. To effectively address colorectal cancer, it is crucial to conduct more research on understudied populations. This will allow us to better assess the connection between genetic variants and predisposition to this disease, as well as gain insights into how these variants may impact patients’ responses to treatment. Investing this area of research can lead to more effective treatment strategies.

## Figures and Tables

**Table 1:** MC: Molecular Consequence, M: Missense, N: Nosense, F: Frameshift, AS: Affecting Splicing, ACMG: ACMG Classification, LP: Likely Pathogenic, P: Pathogenic.

ID	Gene	Variant	MC	rs Identifier	HGVS Nomenclature (GRCh38.p14)	ACMG	Gender	Age Range at DIAGNOSIS (years)	First- and Second-Degree Family History of Cancer
**CC_14**	MUTYH	c.1103G>A (p.Gly368Asp)	M	rs36053993	NC_000001.11: g.45331556C>T	LP	Male	71–75	One parent with colon cancer and another parent with pancreatic cancer, two siblings with colon cancer
**CC_14**	MUTYH	c.452A>G (p.Tyr151Cys)	M	rs34612342	NC_000001.11: g.45332803T>C	LP	* Patient CC_14 as above	* Patient CC_14 as above	* Same patient ID: CC_14 as above
**CC_47**	MUTYH	c.1103G>A (p.Gly368Asp)	M	rs36053993	NC_000001.11: g.45331556C>T	LP	Male	86–90	Parent with colon cancer, sibling with prostate cancer
**CC_69**	MUTYH	c.1103G>A (p.Gly368Asp)	M	rs36053993	NC_000001.11: g.45331556C>T	LP	Female	71–75	None
**CC_03**	MUTYH	c.241C>T (p.Arg81Trp)	M	rs765123255	NC_000001.11: g.45333436G>A	LP	Female	61–65	Parent with colon cancer, sibling with colon cancer
**CC_50**	TP53	c.783-1G>A	AS	rs1555525367	NC_000017.11: g.7673838C>T	LP	Male	81–85	None
**CC_50**	TP53	c.537T>A (p.His179Gln)	M	rs876660821	NC_000017.11: g.7675075A>T	P	* Patient CC_50 as above	* Patient CC_50 as above	* Same patient ID: CC_50 as above
**CC_28**	CHEK2	c.817G>A (p.Glu273Lys)	M	rs587782152	NC_000022.11: g.28710035C>T	P	Male	41–45	None
**CC_54**	CHEK2	c.349A>G (p.Arg117Gly)	M	rs28909982	NC_000022.11: g.28725338T>C	LP	Female	61–65	None
**CC_48**	PALB2	c.1675_1676delCAinsTG (p.Gln559Ter)	N	rs587780206	NC_000016.10: g.23634870_23634871inv	P	Female	31–35	Parent with colon cancer, two siblings with colon cancer
**CC_58**	ATM	c.3994del (p.Ile1332fs)	F	N/A	NC_000011.10: g.108287600del	P	Male	76–80	None
**CC_60**	BARD1	c.176_177del (p.Glu59fs)	F	rs1057517589	NC_000002.12: g.214797104_214797105del	LP	Female	71–75	Parent with prostate cancer

* for emphasizing. N/A: Not applicable

## Data Availability

All data generated or analyzed during this study are included in this published article.
